# Marine Bioactives for Functional Cosmetics with Health-Promoting Properties

**DOI:** 10.3390/md24040143

**Published:** 2026-04-19

**Authors:** Alexandros Tsoupras

**Affiliations:** Hephaestus Laboratory, School of Chemistry, Faculty of Sciences, Democritus University of Thrace, Kavala University Campus, Agios Loukas, 65404 Kavala, Greece; atsoupras@chem.duth.gr

Modern lifestyles expose humans to a dynamic and complex exposome that significantly influences overall health, including skin physiology. Environmental stressors—including ultraviolet (UV) radiation, pollution, poor nutrition, lack of physical activity and increased psychological stress—trigger unfavorable biochemical signaling of oxidative stress and inflammatory pathways that accelerate dermatological issues and contribute to aging complications. While the body activates protective mechanisms, chronic exposure may lead to persistent inflammation, extracellular matrix degradation, and impaired skin function. The concept of the skin exposome encompasses cumulative lifetime exposures that shape skin health. UV radiation remains a primary factor in photoaging and carcinogenesis, while pollutants such as particulate matter, nitrogen dioxide, and ozone, as well as tobacco smoke, exacerbate pigmentation wrinkles and inflammatory skin conditions. Internal factors, including hormonal changes, diet, and stress, further modulate oxidative balance, inflammation, and collagen integrity. Although lifestyle interventions—such as adherence to balanced dietary patterns like the Mediterranean diet, adequate sleep, and daily incorporation of physical activity—support skin health, still, they may not fully counteract the adverse influences of the environmental exposome(s).

In this context, bio-functional cosmetics have emerged as advanced formulations designed to exhibit functional interactions with biological pathways involved in oxidative stress, inflammation, skin barrier function, and microbiome regulation. Within the “clean beauty” movement, there is growing interest in natural bioactive compounds derived from sustainable sources, including plants, microorganisms, animals, and agri-food by-products, often within a sustainability and circular economy framework.

Among these sources, marine-derived bioactives represent one of the most promising and rapidly expanding research fields in the cosmetic sector. The marine environment—characterized by extreme and diverse conditions—drives organisms to produce unique secondary metabolites with potent biological activities. Marine microorganisms, macroalgae, and marine animals constitute a vast and largely untapped reservoir of compounds with multifunctional cosmetic applications.

Marine animals, including fish, mollusks, and crustaceans, and especially their processing by-products, provide valuable biomolecules such as collagen, gelatin, chitin, chitosan, glycosaminoglycans, omega-3 fatty acids, polar lipids, marine phenolics and carotenoids, and bioactive peptides. These compounds exhibit antioxidant, anti-inflammatory, antimicrobial, moisturizing, wound-healing, and anti-aging properties. They support skin regeneration, enhance hydration and elasticity, stimulate collagen synthesis, and protect against UV-induced damage. Importantly, the valorization of marine by-products—such as fish skin, bones, scales, and crustacean shells—aligns with sustainability and circular economy principles, as mandated by the EU and UN, by transforming waste into high-value cosmetic ingredients through sustainable biorefinery approaches.

Equally significant are marine microorganisms and microalgae, which function as efficient biofactories for high-value metabolites. Through advanced fermentation and photobioreactor technologies, these organisms produce carotenoids, polyunsaturated fatty acids, vitamins, phenolics, mycosporine-like amino acids, and bioactive peptides. These compounds contribute to photoprotection, antioxidant defense, microbiome modulation, and anti-aging effects. Microbial-derived ingredients, including probiotics, prebiotics, and postbiotics, further enhance skin barrier function and regulate inflammatory responses, supporting a balanced skin ecosystem. Marine plants, particularly macroalgae (seaweeds), also play a central role as sources of polysaccharides, minerals, and phenolic compounds with hydrating, antioxidant, and protective functions. Collectively, marine-derived ingredients offer exceptional chemical diversity and bioactivity, enabling the development of innovative, multifunctional cosmetic formulations.

Overall, this Special Issue tried to re-emphasize the importance of integrating marine bioresources of natural marine bioactives as important ingredients for bio-functional cosmetics, representing a promising strategy for developing innovative products that not only enhance skin appearance but also support skin health at a biological level, while promoting sustainability and circular economy principles.

Contribution 1: https://doi.org/10.3390/md24030098.

This study investigated the antioxidant, photoprotective, and anti-aging potential of porphyra-334 (PPR-334), a mycosporine-like amino acid (MAA) naturally found in marine organisms, which was biotechnologically produced using *Saccharomyces cerevisiae*. MAAs are well known for their ability to absorb ultraviolet (UV) radiation and protect marine organisms from photooxidative damage, making them promising candidates for cosmetic and dermatological applications. The research focused on developing a large-scale purification process to extract PPR-334 from *Saccharomyces cerevisiae* and on confirming/re-evaluating the biological activities of porphyra-334 in human skin models, particularly its capacity to mitigate UV-induced oxidative stress and cellular damage.

An important aspect of the study is the use of a sustainable biotechnological production system. By engineering *Saccharomyces cerevisiae* to produce porphyra-334, the research provides an alternative to direct extraction from marine organisms, which can be limited by environmental and scalability constraints. This approach aligns with current trends in sustainable and environmentally friendly production of cosmetic ingredients.

Furthermore, the results in human cells showed that porphyra-334 exhibits strong antioxidant activity by scavenging reactive oxygen species (ROS) and increases catalase (CAT) gene expression in human epidermal keratinocyte cells (HEKa). Above all, the authors identified the antioxidant efficacy mechanism of PPR-334 through nuclear factor erythroid 2-related factor 2 (NRF2) and Caspase-9 signals. It was determined that the proliferation efficacy of PPR-334 was due to factors related to the cell cycle. These results demonstrated the anti-aging efficacy of PPR-334 independent of UV irradiation, while enhancing the UV-blocking and antioxidant effects.

In addition to its antioxidant properties, porphyra-334 shows significant photoprotective effects. Its natural UV-absorbing capacity enables it to act as a biological sunscreen, reducing the harmful impact of UV radiation on skin cells. This protective effect extends to the modulation of key molecular pathways involved in photoaging. The study reports that porphyra-334 can regulate the expression of genes and proteins associated with inflammation and extracellular matrix degradation, by suppressing the gene expression of matrix metalloproteinase-1 (MMP-1), known to contribute to collagen breakdown and wrinkle formation. In both HEKa and normal human dermal fibroblast (NHDF) cells, PPR-334 increased collagen expression, and proliferation was also observed in NHDF cells treated with PPR-334, with a simultaneous decrease in advanced glycation end-product (AGE) production. It was confirmed that the efficacy in vitro was also reproduced in human artificial skin tissue models. Thus, PPR-334 was proposed by the authors not only to serve as a sunscreen agent, but also as a dual- or multifunctional material.

Overall, the findings suggest that porphyra-334 is a multifunctional bioactive compound with strong potential for use in bio-functional cosmetics and cosmeceuticals. Its sustainable production from microorganisms, combined with its antioxidant, photoprotective, and anti-aging properties, makes it a promising ingredient for next-generation skincare formulations aimed at protecting against UV-induced damage and promoting long-term skin health.

Contribution 2: https://doi.org/10.3390/md24010003.

This study explored the biological activity and therapeutic potential of a marine-derived protein, Lystar5, isolated from the coelomic cells of *Asterias rubens* starfish, focusing on its effects on human epithelial and immune cells. This protein belongs to the Ly6/uPAR family, a group of molecules known to regulate cell signaling processes related to inflammation, cell migration, and tissue repair. Based on previous outcomes that Lystar5 interacts with nicotinic acetylcholine receptors (nAChRs) and integrin α8-like protein, the research investigated whether Lystar5 and its structurally derived peptide mimetics mediate detachment of coelomic cells from the matrix and their migration, as a model to evaluate whether these proteins can influence cellular behaviors associated with wound healing and tissue regeneration, since skin wound healing in humans is based on keratinocyte migration and is regulated by nAChRs and integrins.

Experimental findings demonstrate that Lystar5 significantly stimulates both individual and collective migration of human skin HaCaT keratinocytes and peripheral blood monocytes, which are essential cells involved in skin repair. Lystar5 can bind to the membrane fraction of coelomic cells with its loops I and II, which form an active site of Lystar5 and resemble its pro-migratory activity. In keratinocytes and monocytes, Lystar5 and the peptides mimicking its loops I and II bound with α3, α4, and β2 nAChR and α5, αV, and β1 integrin subunits, which form molecular complexes. In keratinocytes, Lystar5 and its mimetics promoted short-term E/N cadherin switch and upregulated expression of α5 and αV integrins, EGFR, and ICAM-1. In keratinocytes and monocytes, Lystar5 and its mimetics upregulated E-selectin secretion.

Overall, the ability of Lystar5 and its mimetics to stimulate skin keratinocyte migration and immune cell infiltration may be considered promising for the development of new wound-healing agents. From a broader perspective, this work underscores the significant potential of marine-derived biomolecules as sources of novel functional ingredients for biomedical and cosmetic applications, especially in the development of advanced skin-repairing and bio-functional cosmetic formulations for wound healing.

Contribution 3: https://doi.org/10.3390/md22120532.

This article investigates the anti-melanogenic (skin-whitening) potential of hydroalcoholic extracts and fractions from *Triglochin maritima*, a salt-tolerant plant known for various biological activities, including antioxidant effects, antifungal properties, and skin moisturizing benefits. Different extracts and fractions—70% ethanol extract of *T. maritima* (TME, 10–200 µg/mL) along with its ethyl acetate (TME-EA, 1–15 µg/mL) and water (TME-A, 100–1000 µg/mL) fractions—were prepared and tested on B16F10 melanoma cells stimulated to produce melanin, with or without α-MSH for 72 h. MTT assays were used to assess cytotoxicity, and anti-melanogenesis activity was determined by measuring melanin content, conducting a tyrosinase activity assay, and evaluating the expression of melanogenesis-related genes and proteins via RT-PCR and Western blotting. HPLC-PDA was used to analyze TME and TME-EA.

It was found that TME and especially TME-EA significantly reduced melanin production and tyrosinase activity (a key enzyme in melanin synthesis) in α-MSH-stimulated B16F10 cells, while the water extract showed little effect. Importantly, the most active fraction (TME-EA) demonstrated effects comparable to arbutin, a commonly used skin-whitening agent. Mechanistically, the study showed that the active extract works by down-regulating key genes and proteins involved in melanogenesis (such as Tyr, Mitf, Mc1r, Dct and Trp1) and by reducing CREB, p-38, and JNK phosphorylation while increasing ERK phosphorylation, leading to decreased melanin synthesis, suggesting a significant role for the CREB/MAPK pathway in the observed anti-melanogenic effect of this fraction. Chemical analysis identified luteolin, a flavonoid compound, as a likely major active ingredient responsible for these effects.

The authors suggest that luteolin contributes to the observed antioxidant and enzyme-inhibitory activities, reinforcing its role in suppressing melanogenesis. Overall, the study provides the first evidence that *T. maritima* extracts—particularly the ethyl acetate fraction that is rich in marine flavonoids like luteolin—have promising potential as an effective cosmetic or pharmaceutical agent for hyperpigmentation improvement, due to its anti-melanogenic properties, though further in vivo studies and clinical trials are needed to confirm bio-efficacy and safety.

Contribution 4: https://doi.org/10.3390/md22110503.

This study explored the valorization of the by-products (viscera) from the marine gastropod mollusk *Haliotis discus* (abalone), globally cultivated due to its nutritional value and high market demand, as a sustainable source of marine bioactive hydrolysates and peptides for skin moisturizing and related applications. Various hydrolysates from *H. discus* viscera tissue were evaluated for proximate composition, radical scavenging, and hyaluronidase inhibition activities. In particular, the Alcalase hydrolysate, isolated using gel filtration chromatography (GFC) and assessed for moisturizing effects on human dermal fibroblasts (HDF), HaCaT keratinocytes, and reconstructed human skin tissue, showed the highest extraction yield, as well as antioxidant (radical scavenging) and hyaluronidase inhibition activities. Alcalase hydrolysate GFC fraction 1 significantly enhanced factors associated with skin hydration, as it increased collagen synthesis-related molecules, including procollagen type 1 in HDF and hyaluronic acid-related molecules in HaCaT cells. These moisturizing effects were confirmed in reconstructed human skin tissues by increased levels of aquaporin 3 and filaggrin. Two main peptides, DNPLLPGPPF and SADNPLLPGPPF, were identified in bioactive fraction 1 of the Alcalase hydrolysate of the viscera from *Haliotis discus.*

Overall, the findings highlight the value of marine by-products, such as the viscera from *H. discus*, as sustainable sources of functional ingredients for cosmetics and pharmaceuticals. Abalone viscera hydrolysates—particularly those produced with Alcalase—have promising potential as moisturizing and anti-aging agents. However, further studies, including detailed mechanistic work and clinical validation, are needed before practical applications in cosmeceutical products can be fully applied.

Contribution 5: https://doi.org/10.3390/md23080299.

This article provides a comprehensive review of the valorization of marine animal by-products as sustainable and high-value sources of bioactive compounds with significant potential in health-related and cosmetic applications. It highlights the increasing need to utilize marine processing residues—such as fish skin, bones, scales, mollusk viscera and crustacean shells—within a circular economy framework, transforming waste into valuable functional ingredients.

Marine animal by-products are shown to be rich in diverse biomolecules, including proteins, peptides, polysaccharides, lipids, minerals, and other bioactive metabolites, including amphiphilic bioactives, phenolics, carotenoids, and other pigments. Among the most prominent compounds are collagen, gelatin, chitin, chitosan, glycosaminoglycans, omega-3 fatty acids, polar lipids, phenolics and carotenoids. These molecules exhibit a wide range of biological activities, such as antioxidant, anti-inflammatory, antimicrobial, and tissue-regenerative effects, making them particularly relevant for skin health and cosmetic formulations.

A key focus of the article is the role of these marine-derived compounds in promoting skin regeneration and protection. Marine collagen and gelatin contribute to improved skin hydration, elasticity, and structural integrity, while chitin and chitosan derivatives enhance wound healing, antimicrobial defense, and barrier function. Carotenoids and marine phenolics provide strong antioxidant and photoprotective effects, helping to mitigate oxidative stress and UV-induced damage—two major drivers of skin aging, while bioactive polyunsaturated fatty acids and amphiphilic polar lipids provide strong anti-inflammatory potential, which further protects against aging and other skin inflammatory complications.

The review also emphasizes the importance of bioactive peptides derived from enzymatic hydrolysis of marine proteins. These peptides demonstrate multifunctional properties, including antioxidant, anti-aging, and anti-inflammatory activities, and can modulate cellular pathways associated with collagen, elastin and hyaluronic acid synthesis, the reduction of their breakdown and, thus, extracellular matrix maintenance. Such properties position them as promising ingredients for advanced bio-functional, moisturizing and wound-healing cosmeceutical products.

In addition to their biological activities, the article highlights technological approaches for the efficient extraction and utilization of these compounds. Green extraction methods, enzymatic processes, and marine biorefinery strategies are presented as sustainable solutions that enhance yield, functionality, and environmental performance. These approaches support the transition toward more eco-friendly production systems in the cosmetic and pharmaceutical industries.

Despite their significant potential, challenges remain, including variability in raw materials, scalability of production, regulatory considerations, and the need for further clinical validation. The article underscores the importance of interdisciplinary research and technological innovation to address these limitations and fully exploit marine by-products as bioresources.

Overall, the study concludes that marine animal by-products represent a highly promising, sustainable, and multifunctional source of bioactive compounds. Their integration into bio-functional cosmetics offers a dual benefit: enhancing product efficacy through diverse biological activities while supporting environmental sustainability and waste valorization strategies.

Collectively, the contributions of this Special Issue highlight the marine environment as a highly promising and sustainable reservoir of bioactive compounds for next-generation bio-functional cosmetics and cosmeceuticals. The studies demonstrate that marine-derived ingredients—from microorganisms, marine algae, and plant and animal by-products—exhibit multifunctional biological activities, including antioxidant, anti-inflammatory, photoprotective, antimicrobial, moisturizing and regenerative effects, which directly target key mechanisms of skin health against aging and skin damage.

A central theme across the contributions is the transition from traditional extraction to sustainable biotechnological production, including microbial fermentation and circular economy-driven valorization of marine waste streams. At the same time, advances in formulation technologies, such as nanoencapsulation and targeted delivery systems, are enabling the effective incorporation of sensitive marine bioactives into high-performance cosmetic products.

Importantly, the Special Issue underscores a paradigm shift toward evidence-based, mechanism-driven cosmetic science, supported by molecular studies and emerging clinical validation. Despite existing challenges related to scalability, regulation, and standardization, the integration of biotechnology, green chemistry, and interdisciplinary research is paving the way for innovative, safe, and environmentally responsible skincare solutions.

Overall, the global cosmetic and cosmeceutical market continues to expand rapidly, driven by consumer demand for natural, effective, and sustainable products, with marine-derived bioactives, in particular, gaining increasing attention as next-generation ingredients that combine high performance with environmental responsibility. The contributions of this Special Issue establish marine-derived bioactives as key drivers of innovation in sustainable, high-efficacy cosmetic formulations, with strong potential to shape the future of the global cosmeceutical industry. Future research is expected to focus on interdisciplinary approaches integrating biotechnology, omics technologies, and artificial intelligence to accelerate the discovery and optimization of novel compounds, accompanied by state-of-the-art assays (in vitro, ex vivo and in vivo) that will ensure their safety and bio-efficacy.

